# eL-Chem Viewer: A Freeware Package for the Analysis of Electroanalytical Data and Their Post-Acquisition Processing

**DOI:** 10.3390/s140813943

**Published:** 2014-07-31

**Authors:** Jan Hrbac, Vladimir Halouzka, Libuse Trnkova, Jan Vacek

**Affiliations:** 1 Department of Physical Chemistry, Faculty of Science, Palacky University, 17. listopadu 12, Olomouc 771 46, Czech Republic; E-Mail: vladimirhalouzka@yahoo.com; 2 Department of Chemistry, Faculty of Science, Masaryk University, Kamenice 5, Brno 625 00, Czech Republic; E-Mail: libuse@chemi.muni.cz; 3 Department of Medical Chemistry and Biochemistry, Faculty of Medicine and Dentistry, Palacky University, Hnevotinska 3, Olomouc 775 15, Czech Republic; E-Mail: jan.vacek@upol.cz; 4 CEITEC, Central European Institute of Technology, Brno University of Technology, Technicka 3058/10, Brno 616 00, Czech Republic

**Keywords:** amperometry, sensing, data analysis, data processing, elimination procedure, voltammetry

## Abstract

In electrochemical sensing, a number of voltammetric or amperometric curves are obtained which are subsequently processed, typically by evaluating peak currents and peak potentials or wave heights and half-wave potentials, frequently after background correction. Transformations of voltammetric data can help to extract specific information, e.g., the number of transferred electrons, and can reveal aspects of the studied electrochemical system, e.g., the contribution of adsorption phenomena. In this communication, we introduce a LabView-based software package, ‘eL-Chem Viewer’, which is for the analysis of voltammetric and amperometric data, and enables their post-acquisition processing using semiderivative, semiintegral, derivative, integral and elimination procedures. The software supports the single-click transfer of peak/wave current and potential data to spreadsheet software, a feature that greatly improves productivity when constructing calibration curves, trumpet plots and performing similar tasks. eL-Chem Viewer is freeware and can be downloaded from www.lchem.cz/elchemviewer.htm.

## 1. Introduction

In electrochemical sensing, the analysis of experimental data consists of finding peak or wave heights, positions and other parameters [1]. The appropriate functions are typically included with software controlling electrochemical instrumentation (e.g., BASi, Autolab, CH Instruments), but often only a limited range of options is provided. Several dedicated software packages are available as freeware (e.g., SOAS [2], Tto [3] *etc.*), but these packages are either focused on specific tasks or are text-oriented, requiring knowledge of sets of instructions to perform the desired task, and provide limited graphical output. Taking advantage of the mathematical, graphing and data reporting capabilities of National Instrument's Laboratory Virtual Instrument Engineering Workbench (LabView) we developed a software (‘virtual instrument’) for the transformation and analysis of data obtained from diverse electrochemical experiments. LabView is a system-design platform and development environment for the visual programming language ‘G’. The LabView graphical language logically represents the flow of data, and programmers can view and modify data or control inputs and outputs. Enabling simple interfacing with measurement hardware, the intuitive graphical programming language that is used by LabView is the key to its widespread use with both beginners and experienced programmers in many different scientific fields and industries, including electrochemistry and its application in sensing strategies. To cite a few examples, a LabView-based ‘Virtual electroanalytical instrument for square-wave voltammetry’ received widespread attention [4], a wider range of electroanalytical techniques is covered by the Labview operated instrument described in [5] and a ‘Flexible Software Platform for Fast-Scan Cyclic Voltammetry Data Acquisition and Analysis’ was recently published [6].

In this contribution, we took advantage of LabView's data graphing, signal manipulation and report generation capabilities ito write a software tool ‘eL-Chem Viewer’ for the evaluation and transformation of electrochemical and electroanalytical data. In developing eL-Chem Viewer, emphasis was put on the intuitiveness of its operation.

## 2. Experimental Section

eL-Chem Viewer was written in the LabView 2011 Full Development System with the Report Generation Toolkit add-on (National Instruments, Austin, TX, USA, www.ni.com). To demonstrate individual features of the developed software, the electrochemistry of a potassium ferricyanide standard (1 mM in 0.1 M KCl) was used. To record ferricyanide cyclic, square wave, differential pulse and normal pulse voltammograms, a CHI 660 C electrochemical workstation (CH Instruments, Austin, TX, USA) was used in three-electrode setup, a CHI102 platinum electrode served as the working electrode, CHI111 as the reference and a piece of platinum wire as the auxiliary electrode. The experiments were performed at laboratory temperature (23 °C).

## 3. Results and Discussion

**Figure 1.**
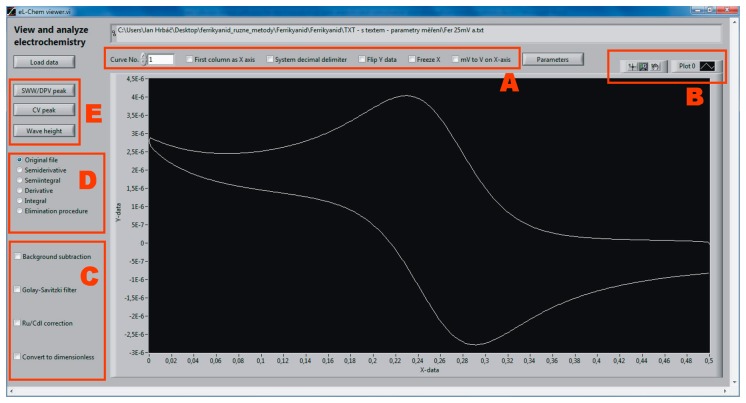
Front panel of eL-Chem Viewer. See above for a description of panels A–E.

The main window of eL-Chem Viewer is shown in Figure 1. After loading and selecting appropriate settings to properly display the data (Figure 1A), the data appear in the graph. Options to change the appearance of the plot as well as useful features such as exporting the graphed data into the clipboard or spreadsheet software are available via *Plot legend* and *Graph palette* (Figure 1B) or the popup menu available by right-clicking in the plot area. Data treatment prior to data transformation and/or analysis can be performed via a selection of checkboxes (Figure 1C). Selecting the desired transformation of the original data is done by selecting from a list of radio buttons (Figure 1D). Peak and wave evaluation routines become available after clicking on the appropriate button (Figure 1E).

### 3.1. Loading the Data Files

A sophisticated algorithm filters off headers and any text between blocks of numeric data, enabling a wide range of variously formatted ASCII data to be accepted—importing Autolab .ocw files, CHI .txt as well as BASi .txt files proceeds with no errors. Files are accepted with spaces, tabs, semicolons as item delimiters and new line (\n) or carriage return (\r) as line delimiters. The setting System decimal delimiter allows the use of the native number format in countries and languages where e.g., a comma is used as the decimal delimiter in numbers. If unchecked, a comma is considered an additional possible item delimiter. In addition to potentiostats' specific ASCII files, two formats of ASCII files containing multi-column XY data are also possible—X1 Y1 X2 Y2 X3 Y3 *etc.* data are accepted by default, the number of the given curve can be selected by the corresponding numeric control. Checking *First column as the X axis* enables the format of an ASCII file containing multiple traces to be changed to X Y1 Y2 Y3 *etc*.

### 3.2. Data Treatment Prior to Data Transformation and/or Data Analysis

#### 3.2.1. Noise Filtering

Noise filtering is accomplished by the Savitzky-Golay algorithm [7], the polynomial order and number of side points is user selectable. The last used combination of parameters is stored and is available the next time the program is started.

#### 3.2.2. Parameter Input

The parameters of a voltammetric experiment (concentration, diffusion coefficient, scan rate, *etc.*), required to perform uncompensated resistance and double-layer capacitance corrections as well as the transformation of voltammograms into dimensionless form (see Sections 3.2.3 and 3.2.4), can be set either globally in the *Parameters* window or locally when performing the desired transformation. In the latter case, only parameters relevant for the desired action are available; other parameters are disabled and grayed. Again, input parameters are stored and are available the next time the program is started.

#### 3.2.3. Uncompensated Resistance and Double Layer Capacitance Correction, Background Subtraction

The use of a potentiostat and working in excess of supporting electrolyte is usually sufficient to obtain voltammograms of acceptable quality. In some cases, especially when working at higher scan rates, a significant distortion of cyclic voltammograms due to uncompensated resistance (*R_u_*) can occur. Common electrochemical workstations allow one to compensate *R_u_* by means of positive feedback or using a current interrupt method, but there are also many instruments in which *R_u_* compensation is not available. Another parameter influencing the shape of voltammogram in an undesired way is the double layer capacitance (*C_DL_*). In the first approximation, *C_DL_* is independent on potential and can be estimated from the initial portion of voltammogram, where no Faradaic reaction occurs [8]. Given that the values of *R_u_* and *C_DL_* are known (*R_u_* can be estimated e.g., using semiintegration, see Section 3.3.1.), mathematical correction can be made for cyclic voltammograms [9]. The potential axis is corrected for IR drop according to Equation (1):

(1) $$E_{t}^{\prime }=E_{t}+I_{t}\cdot R_{u}$$

where *E_t_* is the applied and *E_t_′* the corrected potential (Volts) at a given time point *t*, *R_u_* is uncompensated resistance (Ohms), and *I_t_* is the measured current (Amperes) at a given potential point in the voltammogram. The current axis is corrected according to Equation (2):

(2) $$I_{F,t}=I_{t}+C_{\text{DL}}\frac{dE_{t}^{\prime }}{\text{dt}}+R_{u}C_{\text{DL}}\frac{{\text{dI}}_{t}}{\text{dt}}$$

where *I_F,t_* is the Faradaic (*i.e.*, uncompensated resistance and double layer capacitance corrected) current and *C_DL_* is the double-layer capacitance in Farads. The resulting corrected voltammogram is *I_F,t_ vs. E_t_′* plot; after the correction, the voltammogram is interpolated to achieve a uniform spacing of the X-data. As an alternative to double-layer capacitance correction, a *background subtraction* of a blank voltammogram can be used. In this case, the X-data of both voltammograms should match. Separate uncompensated resistance correction can be made afterwards, by setting the value of the *Double layer capacitance* parameter to zero.

#### 3.2.4. Conversion of Voltammograms into Dimensionless Form

The conversion of cyclic voltammograms into dimensionless form is advantageous when performing semiintegration (see below). The conversion is based on the application of Equation (3) to voltammetric data:

(3) $$\Psi \left(t\right)=\frac{I_{t}}{\text{FAC}\sqrt{\frac{\text{DFv}}{\text{RT}}}}$$

where *ψ*(*t*) is the dimensionless current, *I_t_* is the current (Y-) data in studied voltammogram (Amperes), *F* is the Faraday constant (C·mol^−1^), *A* electrode area (cm^2^), *C* concentration (mol·cm^−3^), *D* diffusion coefficient (cm^2^·s^−1^), *v* scan rate (V·s^−1^), *R* universal gas constant (J·K^−1^·mol^−1^) and *T* thermodynamic temperature (K). When the convolution (semiintegration) is performed on dimensionless data, the plateau current corresponds to the apparent number of electrons passed in the redox process. This conclusion assumes that the entered values of concentration, diffusion coefficient, electrode area, *etc.* are correct.

### 3.3. Data Transformations

#### 3.3.1. Semiderivative and Semiintegral Convolution, Derivative and Integral

Semiintegration [10] is a special case of convolution used in voltammetry, the voltammograms are transformed into a wave-shaped response, often denoted a ‘neopolarogram’ [11,12]. Semidifferentiation is achieved by semiintegration and subsequent differentiation of the studied voltammogram. It is useful in the voltammetric stripping procedure to achieve sharp, easy-to-evaluate peaks and can be used to increase the peak separation of overlapped voltammetric responses [13,14]. Semiintegration (and semidifferentiation) can facilitate the evaluation of voltammetric data namely by its insensitivity to electrode kinetics, enabling the number of transferred electrons to be evaluated, as outlined in the previous section.

Another widespread use is in the evaluation of diffusion coefficients. The superiority of the convolution approach over steady-state (hydrodynamic or microelectrode) voltammetric techniques to evaluate diffusion coefficients in viscous ionic liquids is demonstrated in [15]. Among other uses of semiintegration is an estimation of uncompensated resistance, detectable as the deviation from congruence of the forward and backward scans of the neopolarogram [16]. Semiintegration and semidifferentiation is also a very useful tool for determining adsorption coupled to electrochemical processes [17].

#### 3.3.2. Elimination Voltammetric Procedure

**Figure 2.**
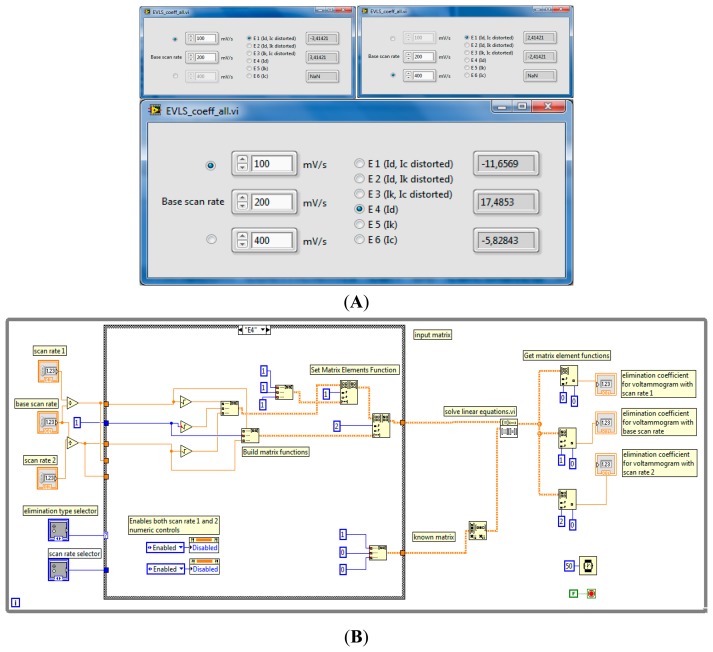
(**A**) Front panel of elimination coefficient calculator subroutine, upper left: calculation of elimination coefficients to eliminate kinetic current from the first two voltammograms and (upper right) second two voltammograms with indicated scan rate values. Below: calculation of elimination coefficients leaving only the diffusion current (Id) for the whole trinity of voltammograms; (**B**) Elimination coefficients' calculator subVI. Annotated block diagram.

A less known, but useful transformation of voltammetric data is the elimination technique [18]. The elimination procedure enables the removal of current components which are proportional to the zeroth, square root and first power of the scan rate from the voltammogram. This corresponds to the removal of capacitive, diffusion and kinetic current components [19], provided that only the aforementioned components are present in the voltammogram. The algorithm implemented in eL-ChemViewer assumes that uncompensated resistance is negligible or corrected and planar diffusion applies exclusively (for elimination procedure under the conditions of radial diffusion, refer to [20]). To achieve elimination, besides the studied voltammogram one (when only one of the three current components is being removed‐‘eliminated’) or two (two current components are eliminated) additional voltammograms are required to perform the linear combination using specific coefficients (further denoted as elimination coefficients). The voltammograms entering the elimination procedure differ in their scan rates. Elimination coefficients can be calculated [21] using dedicated mathematical software packages (*i.e.*, Matlab, Maple *etc.*) and the transformation of voltammograms using the obtained coefficients can be subsequently performed in spreadsheet software. The general unavailability of mathematical software in electrochemical workplaces and the rather specialized knowledge required to operate these packages limits the availability of the generalized elimination technique to several laboratories. As a result, a special case [22–25] is used more often, in which the scan rates are in the ratio of 0.5*v*:1*v*:2*v* (*v* is the ‘base’ or ‘reference’ scan rate). In this case, the linear combination in which only diffusion component remains in the reversible voltammogram is (Equation (4)):

(4) $$f(I)=-11.657I_{1/2}+17.485I-5.8284I_{2}$$

where *I*_1/2_, *I* and *I*_2_ are the Y-data of voltammograms corresponding to scan rates of 0.5 *v*, 1 *v* and 2 *v*, respectively (e.g., 0.1, 0.2 and 0.4 V·s^−1^). When this equation is applied to a trinity of voltammograms in which the dominant electrode process is not diffusion, but the electroactive substance being adsorbed onto the surface of the electrode before an electron transfer, this elimination provides a unique peak-counter peak signal, making a rapid determination of adsorption possible [26]. The LabView routine, the front panel of which is shown in Figure 2A enables elimination coefficients to be calculated for the given elimination type (E1–E6, this notation is used in the associated literature (e.g., [26]) and any combination of scan rates. The calculation is performed for two (in case of E1–E3 elimination functions) or three arbitrary scan rates (E4–E6 elimination functions). For eliminations requiring two scan rate values, radio buttons enable selection of the upper or lower scan rate numeric control, the remaining (third) numeric control is disabled.

For readers interested in LabView programming, the block diagram of the routine for the case of E4 elimination is shown in Figure 2B. The set of LabView functions enabling work with matrices is used. The input and known matrices are created for each elimination function from the user data, this user data having been entered into the corresponding scan rate numeric controls and supplied into the inputs of the ‘Solve Linear Equations.vi’ LabView function. The elements of the solution matrix contain resulting elimination coefficients which are displayed in the corresponding numeric indicators.

**Figure 3.**
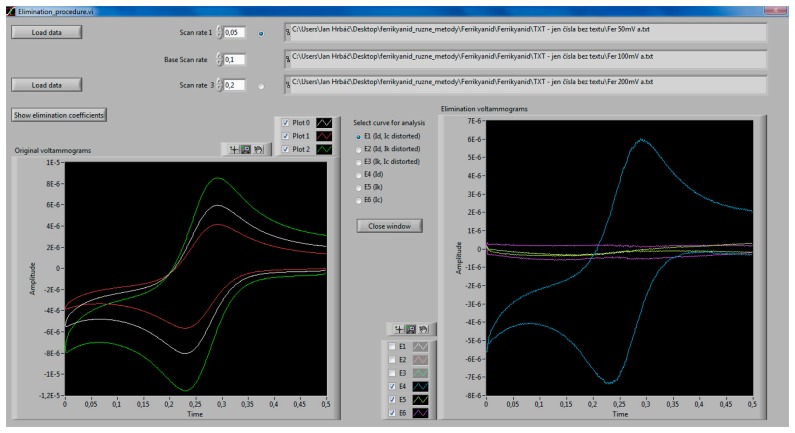
Elimination procedure dialog window, showing three ferricyanide voltammograms used for elimination (left graph) and voltammograms processed by elimination showing the diffusion, kinetic and capacitive partial currents (blue, yellow, resp. violet traces in the right graph).

The routine described above is the basis for the elimination procedure implementation in eL-Chem Viewer. When choosing *Elimination procedure* in the main window, the file is copied into a newly opened elimination procedure window (Figure 3) as a voltammogram recorded at the base (reference) scan rate. The required additional voltammograms are loaded by the user and the corresponding scan rate data entered. When the base voltammogram has been manipulated by smoothing, background corrected or *C_DL_*/*R_u_* compensated (Equations (1) and (2)), the same treatment automatically applies for the additional voltammograms. Six different elimination voltammograms are obtained and can be viewed in the graph. After selecting the desired curve by radio button and closing the window, the chosen elimination voltammogram can be further processed.

### 3.4. Evaluation of Peaks & Waves, Cooperation with Spreadsheet Software

**Figure 4.**
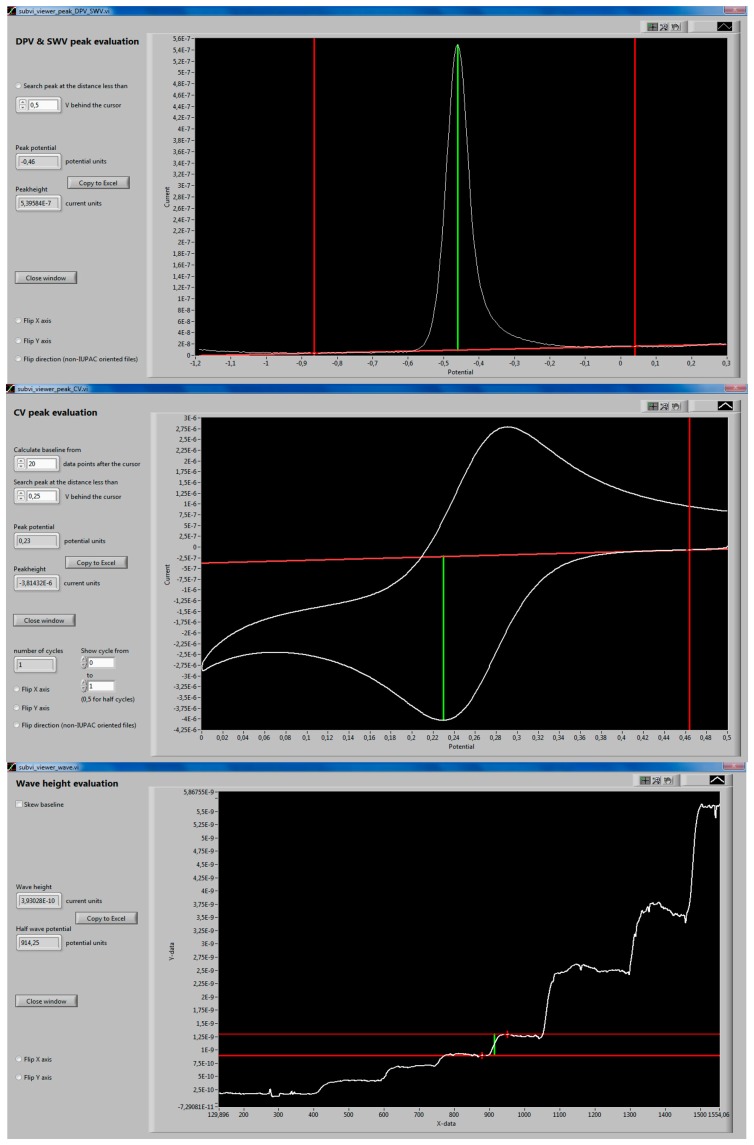

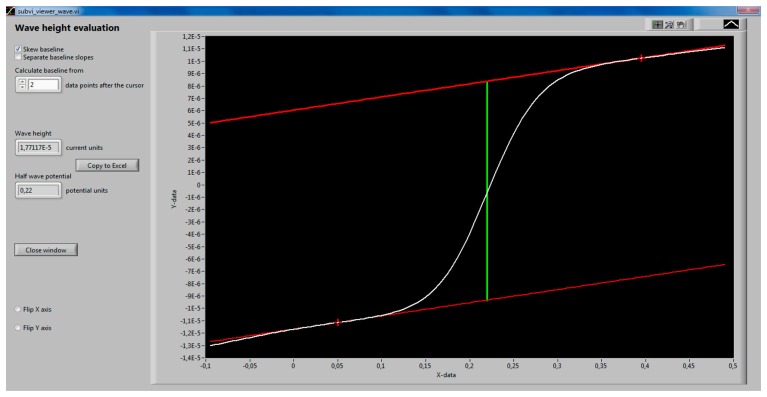
Peaks and waves evaluation subroutines' front panels.

DP and square-wave voltammograms are evaluated by setting up the baseline using the cursors positioned as shown in Figure 4. Peak position is automatically found and indicated by the green line provided that IUPAC-oriented voltammograms are evaluated. When the voltammograms are not IUPAC-oriented, the checkbox allowing the evaluation of non-IUPAC-oriented files must be selected. Unresolved peaks and shoulders can be evaluated by restricting the distance within which the peak is searched for (*i.e.*, the cursor—peak distance). After finding the peak, the obtained values of peak currents and potentials can be imported into spreadsheet software (MS Excel) by clicking the *Copy to Excel* button. When the button is first clicked, the Excel program opens, a new worksheet is created and potential/current data is transferred into a row, potential value as the first item. The cursor is then set to a new line and the worksheet is ready to accept the next set of data.

Cyclic voltammograms are evaluated in a similar manner to DP and SW voltammograms, the difference is that the baseline is defined by the tangent to the voltammogram at the cursor position. To calculate the tan\n gent, linear regression of the portion of the curve at the position of the cursor(s) is made, the length of this portion(s) can be set by the user. For multiple cycles' containing voltammograms, the software finds the number of CV cycles and allows the selected part of voltammograms to be viewed, the minimum displayable section is one half cycle.

DC polarography, rotating disk electrode voltammetry, microelectrode voltammetry, but also voltammetric measurement transformed by semiintegration are all experiments that produce wave-shaped response curves. Another example of this type of response are the staircase-like response curves from sensors and biosensors, usually obtained from constant potential amperometry in stirred solutions, into which aliquots of the tested analyte are introduced manually or by using an auto sampler. To evaluate waves, three modes of operation are available. A flat baseline is usually convenient to evaluate amperograms. In *skewed baseline* mode, which is appropriate e.g., for NPV, a (default) single slope mode means that one of the baselines (plotted in a thicker line) is skewed to match the slope of the tangent at the point of the curve indicated by the cursor. The second baseline adopts the slope of the first one. In *separate baseline slopes* mode, the baselines are independent of each other.

## 4. Conclusions

Here we have introduced eL-ChemViewer, a LabView-based freeware package for the analysis of voltammetric and amperometric data, and their post-acquisition processing using semiderivative, semiintegral, derivative, integral and elimination procedures. The software supports the single-click transfer of peak/wave current and potential data to spreadsheet software, a feature that greatly improves productivity when constructing calibration curves, trumpet plots and performing similar tasks. The eL-Chem Viewer installer can be downloaded at www.lchem.cz/elchemviewer.htm. The software can be used freely if properly cited according to the information on the web page.
